# Design of CO_2_-Resistant High-Entropy Perovskites Based on Ba_0.5_Sr_0.5_Co_0.8_Fe_0.2_O_3-δ_ Materials

**DOI:** 10.3390/ma17184672

**Published:** 2024-09-23

**Authors:** Yongfan Zhu, Jia Liu, Zhengkun Liu, Gongping Liu, Wanqin Jin

**Affiliations:** State Key Laboratory of Materials-Oriented Chemical Engineering, College of Chemical Engineering, Nanjing Tech University, No. 30 Puzhu Road (S), Nanjing 211816, China; 202262104053@njtech.edu.cn (Y.Z.);

**Keywords:** high-entropy perovskite, CO_2_ resistance, membrane, oxygen permeability

## Abstract

High-entropy perovskite materials (HEPMs), characterized by their multi-element composition and highly disordered structure, can incorporate multiple rare earth elements at the A-site, producing perovskites with enhanced CO_2_ resistance, making them stay high performance and structurally stable in the CO_2_ atmosphere. However, this modification may result in reduced oxygen permeability. In this study, we investigated La_0.2_Pr_0.2_Nd_0.2_Ba_0.2_Sr_0.2_Co_0.8_Fe_0.2_O_3-δ_ (L_0.2_M_1.8_) high-entropy perovskite materials, focusing on enhancing their oxygen permeability in both air and CO_2_ atmospheres through strategic design modifications at the B-sites and A/B-sites. We prepared Ni-substituted La_0.2_Pr_0.2_Nd_0.2_Ba_0.2_Sr_0.2_Co_0.7_Fe_0.2_Ni_0.1_O_3-δ_ (L_0.2_M_1.7_N_0.1_) HEPMs by introducing Ni elements at the B-site, and further innovatively introduced A-site defects to prepare La_0.2_Pr_0.2_Nd_0.2_Ba_0.2_Sr_0.2_Co_0.7_Fe_0.2_Ni_0.1_O_3-δ_ (L_0.1_M_1.7_N_0.1_) materials. In a pure CO_2_ atmosphere, the oxygen permeation flux of the L_0.1_M_1.7_N_0.1_ membrane can reach 0.29 mL·cm^−2^·min^−1^. Notably, the L_0.1_M_1.7_N_0.1_ membrane maintained a good perovskite structure after stability tests extending up to 120 h under 20% CO_2_/80% He atmosphere. These findings suggest that A-site-defect high-entropy perovskites hold great promise for applications in CO_2_ capture, storage, and utilization.

## 1. Introduction

The combustion of fossil fuels produces a significant amount of CO_2_, contributing to the greenhouse effect [1,2]. CO_2_ capture and storage technology (CCS) is considered one of the most promising solutions to mitigate this issue [3,4]. Using pure oxygen instead of air in the oxygen-enriched combustion process allows for nitrogen-free combustion of fossil fuels, facilitating the separation and storage of high-concentration CO_2_ from flue gas [5]. Dense ceramic oxygen permeation membranes based on perovskite materials, with their theoretical 100% selectivity, can achieve oxygen permeation without an external circuit [6]. This makes them ideal for producing high-purity oxygen. The high-purity oxygen separated by these membranes can be utilized for fossil fuel combustion in coal-fired power plants. Additionally, part of the CO_2_ produced can be recycled as a purge gas and heat source for the oxygen permeation membrane [7]. However, beyond the requirement for oxygen permeation, thermochemical stability and stable performance under operating conditions, particularly in CO_2_ atmospheres, are critical factors limiting the industrial application of these membranes [8,9]. Ensuring both high oxygen permeability and stability in CO_2_ environments is prerequisite for their successful deployment in CCS technologies.

In recent years, “high entropy” has emerged as a popular material design theory in material research. High-entropy materials usually contain five or more elements. Due to their high configurational entropy and disordered atomic structure, high-entropy materials exhibit unique “four major effects”, which are the high-entropy effect, lattice distortion effect, sluggish diffusion effect, and “cocktail” effect. These unique effects enhance the strength, corrosion resistance, and thermal stability of high-entropy materials. Researchers have extended the high-entropy alloy design concept to ceramic materials, leading to the development of various high-entropy ceramic systems, including high-entropy oxides [10,11], nitrides [12,13,14], and borides [15,16,17]. These systems offer new possibilities for creating multifunctional materials. A novel approach is required to develop perovskite oxygen permeation membrane materials resistant to CO_2_ atmospheres. Instead of traditional single-component metal element doping at the A- or B-site, co-doping with multiple metal elements in perovskite materials can yield high-performance oxygen permeation membranes [18,19,20,21]. This strategy leverages the benefits of high entropy to enhance both oxygen permeability and CO_2_ resistance [22,23], providing a promising pathway for advanced material development.

From the perspective of crystallographic doping design, the high-entropy design of perovskite materials can be approached from three directions: multi-element doping at the A-site, such as Ca_0.1_La_0.02_Gd_0.02_Bi_0.02_Ba_0.42_Sr_0.42_Co_0.8_Fe_0.2_O_3-*δ*_ [21], multi-element doping at the B-site, such as Ba_0.5_2Sr_0.5_Co_0.736_Fe_0.184_Zr_0.02_Ni_0.02_Cu_0.02_Al_0.02_O_3-*δ*_, and multi-element doping at both A- and B-sites, such as Ca_0.1_La_0.02_Gd_0.02_Bi_0.02_Ba_0.42_Sr_0.42_Co_0.736_-Fe_0.184_Zr_0.02_Ni_0.02_Cu_0.02_ Al_0.02_O_3-*δ*_ [21]. Depending on the specific elements introduced, the material’s stability under various conditions can be enhanced [10,24,25]. However, the introduced elements often have single oxidation states (e.g., La, Pr, and Bi at the A-site) [26,27] or high oxidation states (e.g., Ta, Nb, and Al at the B-site) [25,28], resulting in lower oxygen permeability in the high-entropy perovskite material compared to its parent material. Improving oxygen permeability while maintaining the stability of high-entropy materials is crucial to address this issue. A common strategy involves introducing a single multi-valent and easily variable transition metal at the B-site and designing defects at the A-site [29,30,31,32,33]. These methods can optimize oxygen permeability performance and mitigate the reduced performance typically observed in perovskite materials after high-entropy design.

In this study, Ba_0.5_Sr_0.5_Co_0.8_Fe_0.2_O_3-δ_ (BSCF) materials, known for their high oxygen flux, were utilized as the matrix for implementing high-entropy CO_2_-resistant design at the A-site. To reduce the potential decrease in oxygen permeability of the high-entropy CO_2_-resistant design, Ni with multi-valent and easily variable transition was substitution at the B-site. In addition, an A-site defect was performed to increase the oxygen permeability of the high-entropy CO_2_-resistant design. Theoretical calculations were performed to obtain the Goldschmidt tolerance factor, cation size difference, and mixing entropy value of the material. Based on the feasibility indicated by these calculations, the high-entropy perovskite material La_0.2_Pr_0.2_Nd_0.2_Ba_0.2_Sr_0.2_Co_0.8_Fe_0.2_O_3-δ_ (L_0.2_M_1.8_) was prepared using the solid-state reaction method. Further modifications included Ni substitution at the B-site and defect design at the A-site of the L_0.2_M_1.8_ material, named La_0.2_Pr_0.2_Nd_0.2_Ba_0.2_Sr_0.2_Co_0.7_Fe_0.2_Ni_0.1_O_3-δ_ (L_0.2_M_1.7_N_0.1_) and La_0.1_Pr_0.2_Nd_0.2_Ba_0.2_Sr_0.2_Co_0.7_ Fe_0.2_Ni_0.1_O_3-δ_ (L_0.1_M_1.7_N_0.1_). This work systematically studied the crystal structure, micromorphology, X-ray photoelectron spectroscopy, and carbon dioxide adsorption and desorption properties of the synthesized high-entropy perovskite oxide. Additionally, the relationship between the oxygen permeation flux of the HEPMs and variables such as temperature and CO_2_ concentration were investigated, as well as stability.

## 2. Materials and Methods

The perovskite powder was synthesized by the solid-phase reaction method. Doping at the A-site and B-site was determined by adjusting the molar ratios of the raw materials. The A-site and B-site doping ratios were precisely regulated according to stoichiometric principles. A specified mass of La_2_O_3_ (aladdion, Shanghai, China, AR, 99.99%), Pr_2_O_3_ (MACKLIN, Shanghai, China, metal basis, 99.9%), Nd_2_O_3_ (MACKLIN, metal basis, 99.9%), BaCO_3_ (Sinopharm Chemical Reagent Co., Ltd., Shanghai, China, SP, ≥99%), SrCO_3_ (Sinopharm Chemical Reagent Co., Ltd., SP, ≥99%), Co_2_O_3_ (Sinopharm Chemical Reagent Co., Ltd., SP, ≥99%), Fe_2_O_3_ (Sinopharm Chemical Reagent Co., Ltd., SP, ≥99%), and NiO (aladdion, AR, 99%) powders were weighed according to the stoichiometric ratio, using ethanol as the ball milling medium. A uniformly mixed raw material powder was obtained after ball milling, drying, and sieving. Subsequently, three high-entropy perovskite powders were produced by calcining the mixture at 1100 °C for 5 h.

The dense disk membranes were prepared by isostatic pressing. Using Polyvinyl alcohol (PVA) as a binder, the perovskite powder and PVA were evenly ground and transferred to a specific mold, and then pressed into a membrane precursor at a pressure of 15 MPa. The dense perovskite ceramic oxygen-permeable membrane was obtained by heating to 1230 °C at a rate of 2 °C/min in a muffle furnace and maintaining this temperature for 5 h. For simplicity, the La_0.2_Pr_0.2_Nd_0.2_Ba_0.2_Sr_0.2_Co_0.8_Fe_0.2_O_3_, La_0.2_Pr_0.2_Nd_0.2_Ba_0.2_Sr_0.2_Co_0.7_Fe_0.2_ Ni_0.1_O_3-δ_, and La_0.1_Pr_0.2_Nd_0.2_Ba_0.2_Sr_0.2_Co_0.7_ Fe_0.2_Ni_0.1_O_3-δ_ samples are referred to as L_0.2_M_1.8_, L_0.2_M_1.7_N_0.1_, and L_0.1_M_1.7_N_0.1_, respectively. Oxygen permeation tests were conducted using homemade oxygen permeation components, with GC-7820 on-line gas chromatography.

The crystal structure of the membrane was characterized using X-ray diffraction (XRD, Rigaku Smart Lab 9KW, Akishima, Japan) with Cu Kα radiation (wavelength: 1.542 Å) and a test step of 0.02°. Microscopic morphology was examined using a TM3000 desktop scanning electron microscope (Hitachi, Tokyo, Japan), and the corresponding Energy Dispersive Spectrometer (EDS, AztecLiveOne, Oxford, UK) was used to confirm the presence of each element in the perovskite oxides. To enhance sample conductivity, the samples were coated with gold using an ion sputtering coater (LDM-150D, Shanghai Huayan, Shanghai, China). Temperature-programmed desorption (TPD) tests for oxygen and carbon dioxide were performed using a fully automatic chemisorption instrument (BELCAT-II, Microtrac BEL, Osaka, Japan). The elemental composition and valence state of the surface of the high-entropy perovskite powders were analyzed using X-ray photoelectron spectroscopy (XPS, Thermo ESCALAB 250, Waltham, MA, USA). Charge correction was performed using the standard peak position of the C 1s binding energy at 284.8 eV.

### Formatting of Mathematical Components

High-entropy perovskite oxides typically comprise five or more metal cations at the A- or B-sites. The ionic radii of the various elements are different, which may cause lattice distortion effects on the structure. In the high-entropy design process, it is crucial to thoroughly consider the impact of the differences in the ionic radii of various cations on the stability of the perovskite phase structure. The stability of perovskite oxides can be assessed using three key parameters: (1) The Goldschmidt tolerance factor (t), which is a parameter used to predict the stability and formability of perovskite structures. (2) The size difference of the cations at the A-site (*δ*(*R*_*A*_)) and B-site (*δ*(*R*_*B*_)), which refers to the disparity in ionic radii between the A-site and B-site cations in a perovskite structure. (3) The mixing entropy (ΔS_mix_), which is used to determine whether the material is a high-entropy material. These parameters can be calculated using Equations (1), (2), (3), and (4), respectively:

(1) $$t=\frac{r_{A}+r_{O}}{\sqrt{2}\left(r_{B}+r_{O}\right)}$$

where $r_{A}$, $r_{B}$, and $r_{O}$ are the average ionic radii of A- and B-sites and oxygen, respectively.

(2) $$\delta \left(R_{A}\right)=\sqrt{\sum_{i=1}^{N}c_{i}{\left(1-\frac{R_{A_{i}}}{\left(\sum_{i=1}^{N}c_{i}R_{A_{i}}\right)}\right)}^{2}}$$

where $R_{A_{i}}$ and $c_{i}$ are the cation radius and corresponding molar fraction of the A-site element, respectively.

(3) $$\delta \left(R_{B}\right)=\sqrt{\sum_{i=1}^{N}c_{i}{\left(1-\frac{R_{B_{i}}}{\left(\sum_{i=1}^{N}c_{i}R_{B_{i}}\right)}\right)}^{2}}$$

where $R_{B_{i}}$ and $c_{i}$ are the cation radius and corresponding molar fraction of the B site element, respectively.

(4) $${\Delta S}_{mix}=-R\left[{\left(\sum_{a=1}^{n}x_{a}\;lnx_{a}\right)}_{A-site}+{\left(\sum_{b=1}^{n}x_{b}\;lnx_{b}\right)}_{B-site}+3{\left(\sum_{c=1}^{n}x_{c}\;lnx_{c}\right)}_{O-site}\right]$$

where $x_{a}$, $x_{b}$, and $x_{c}$ are the molar fractions of metal ions at the A- and B- sites and oxygen, respectively.

## 3. Results

Table 1 presents the calculated values for the tolerance factor (t), cation size difference (δ), and mixing entropy (ΔS_mix_) of three high-entropy perovskite materials based on their respective cation radii and molar fractions.

As shown in Table 1, the tolerance factor (t) values for the three high-entropy perovskite materials fall within the stability range for perovskite formation (0.78 < t < 1.05), supporting the theoretical feasibility of synthesizing single-phase high-entropy perovskite oxides. In the design of high-entropy alloys, achieving a single-phase structure typically requires an atomic size difference (δ) of less than 6.5% [34]. However, for stable high-entropy perovskite oxides, the acceptable δ range is generally 6% to 12% [23,34]. The calculated δ values for the A-sites of materials L_0.2_M_1.8_, L_0.2_M_1.7_N_0.1_, and L_0.1_M_1.7_N_0.1_ are 8.48%, 8.48%, and 8.49%, respectively, aligning with the criteria for single-phase high-entropy oxide formation. Additionally, the formation of high-entropy perovskites necessitates a mixing entropy (ΔS_mix_) greater than 1.5R (R = 8.314 J·K^−1^·mol^−1^) [35,36], which is satisfied by the ΔS_mix_ values of all three materials. Consequently, these findings confirm the potential to synthesize single-phase high-entropy perovskite oxides.

**Figure 1:**
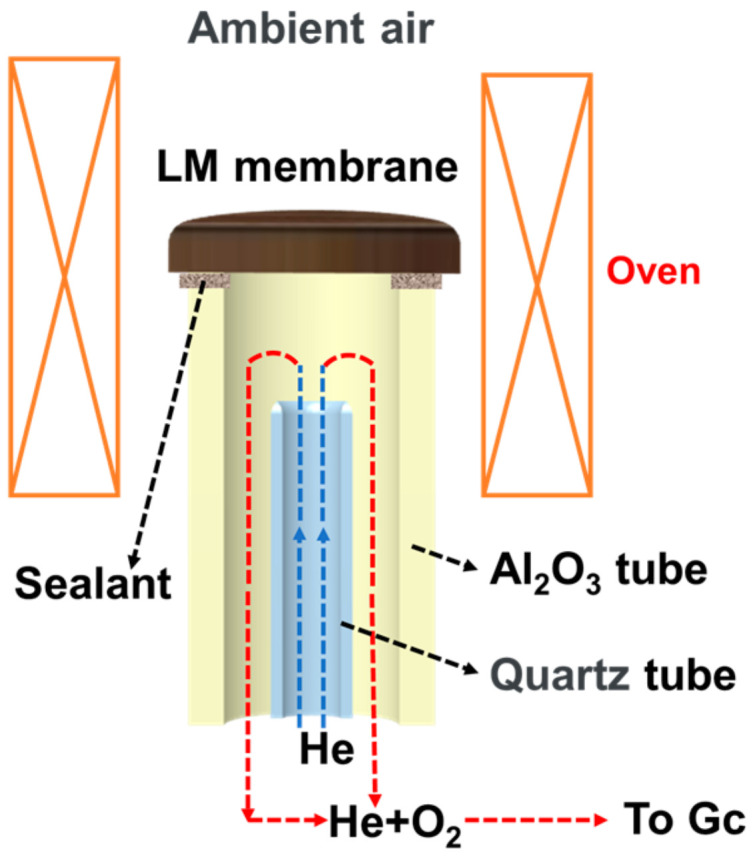
The schematic of the oxygen permeation test apparatus.

Figure 1 shows the schematic of the oxygen permeation test apparatus. The oxygen permeation tests of three materials and the long-time test of L_0.1_M_1.7_N_0.1_ were conducted using this apparatus.

**Figure 2:**
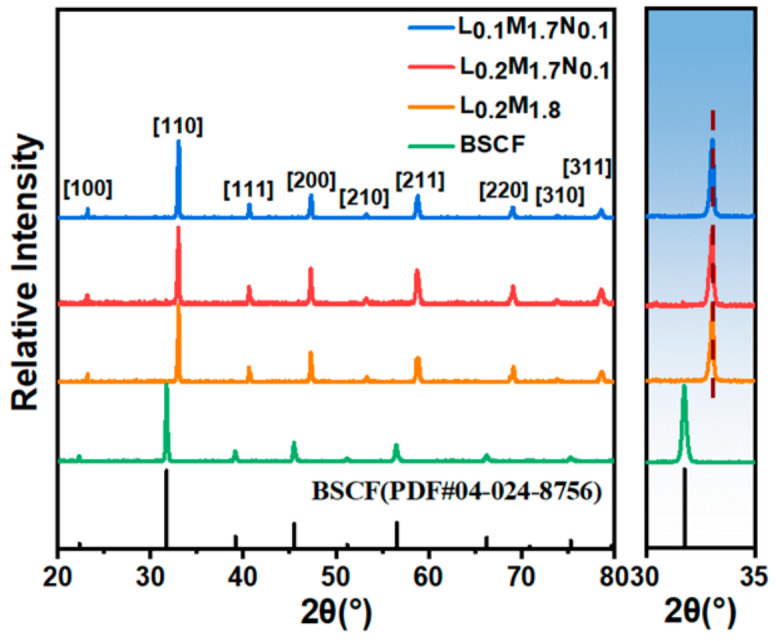
XRD diffraction patterns of the high-entropy perovskite oxide membranes.

Figure 2 shows the XRD diffraction patterns of the membranes. The diffraction patterns of the BSCF parent material and the three high-entropy perovskite films all exhibit characteristic peaks of perovskite oxides.

**Figure 3:**
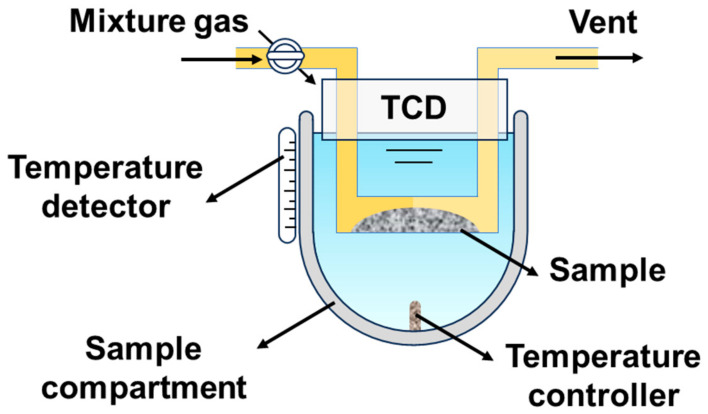
The schematic of the chemical desorption test system.

Figure 3 shows the schematic of the chemical desorption test system. The O_2_ and CO_2_ temperature-programmed desorption of all three materials were conducted using this system.

**Figure 4:**
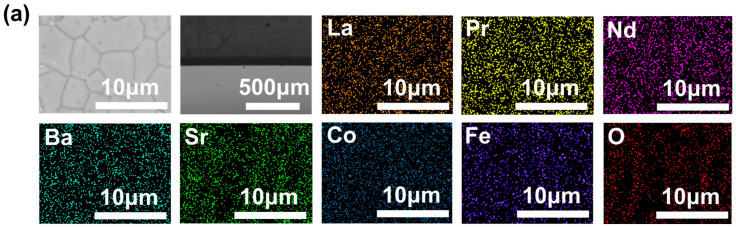

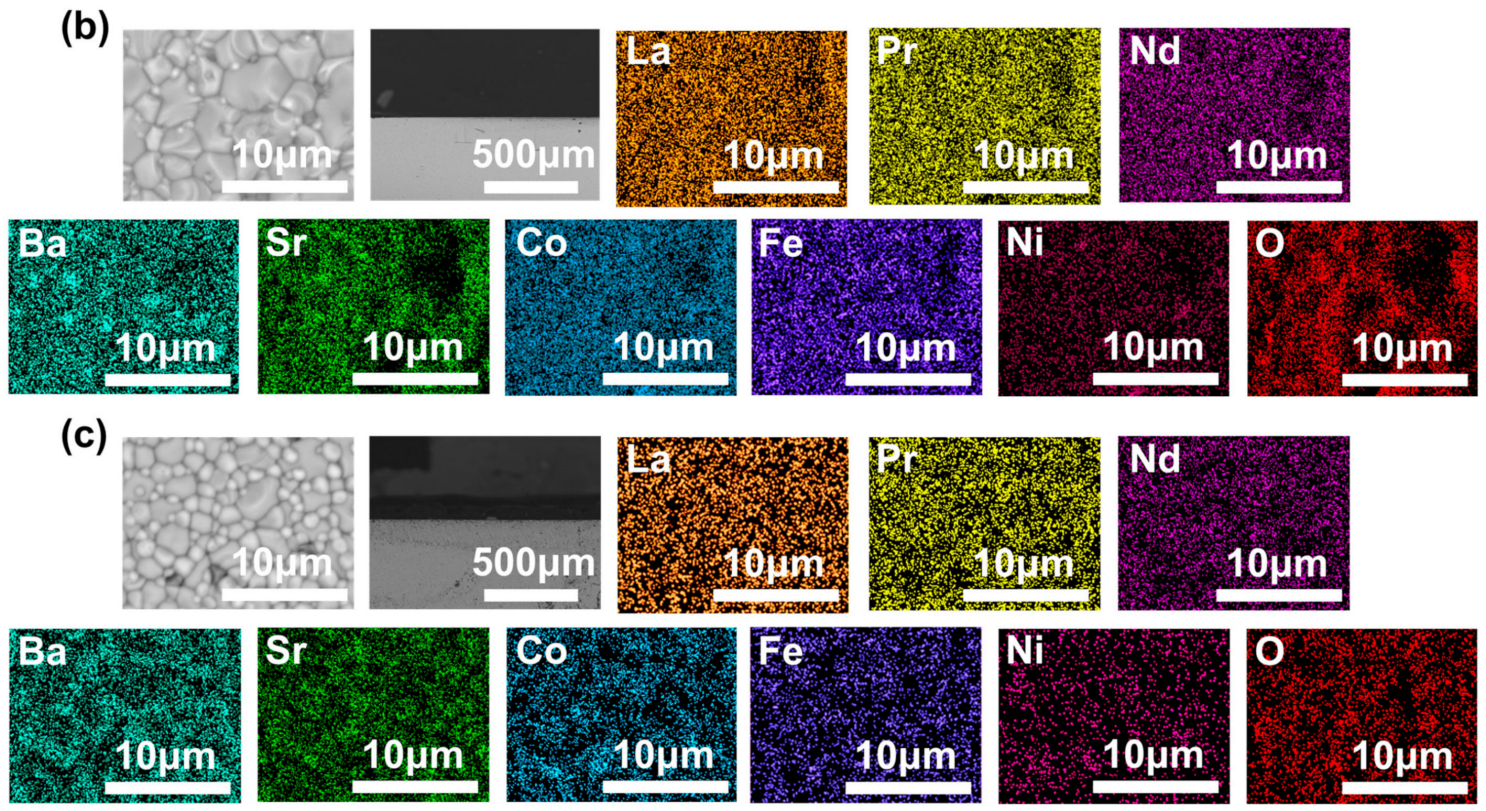
SEM images of the disk membrane surface and cross-section: (**a**) L_0.2_M_1.8_; (**b**) L_0.2_M_1.7_N_0.1_; and (**c**) L_0.1_M_1.7_N_0.1_.

The SEM characterization results in Figure 4 reveal that the surface of the membrane is dense and has no intra-grain defect. The cross-sectional structure shows that the cross-sections of all three membranes are uniformly dense and devoid of through-holes. The corresponding EDS data exhibit that all elements were evenly distributed in the membranes.

**Figure 5:**
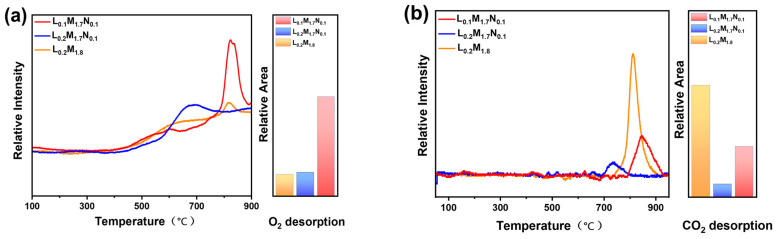
Temperature-programmed desorption curves and relative desorption peak areas of high-entropy perovskite oxide powders (**a**) O_2_-TPD and (**b**) CO_2_-TPD.

The results of the temperature-programmed desorption test are shown in Figure 5. It can be seen from Figure 5a that the oxygen desorption peaks of the HEPMs are mainly concentrated in the high-temperature zone. The carbon dioxide adsorption and desorption performance of HEPMs are presented in Figure 5b. At low temperatures, the three materials have almost no desorption effect on CO_2_. However, significant differences in the CO_2_ desorption peaks are observed at higher temperatures among the HEPMs.

**Figure 6:**
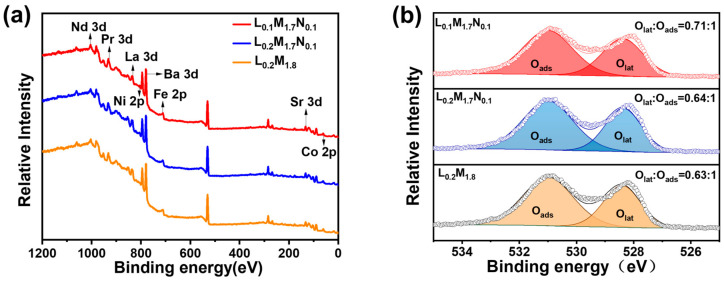
(**a**) XPS spectra of the HEPMs membrane; (**b**) XPS spectra of O 1s peaks.

The HEPM membranes were analyzed using X-ray photoelectron spectroscopy (XPS), with the results presented in Figure 6. All elements in the membranes were detected. Figure 6b shows the XPS spectra of the three samples. The low binding energy peaks correspond to lattice oxygen (O_lat_), while the high binding energy peaks correspond to adsorbed oxygen (O_ads_).

**Figure 7:**
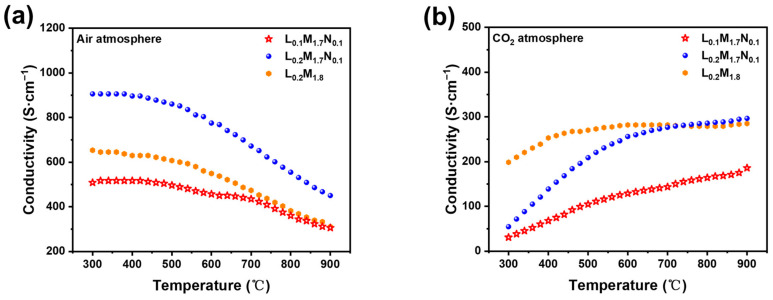
Temperature dependence of the total conductivity of the high-entropy perovskite: (**a**) air atmosphere; (**b**) CO_2_ atmosphere.

Figure 7a,b illustrate the conductivity of the three kinds of strip membranes in air and CO_2_ atmospheres as a function of temperature. As shown in Figure 7a, the electric conductivity of the HEPMs in the air atmosphere decreases with increasing temperature, showing metal-like conductive behavior. Figure 5b shows the conductivity of three kinds of strip membranes in a CO_2_ atmosphere as a function of temperature.

**Figure 8:**
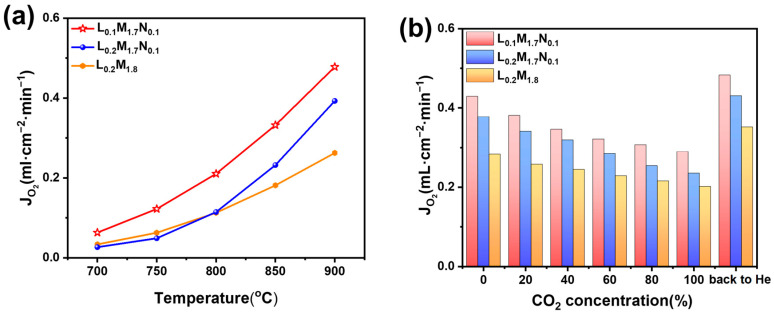
(**a**) Oxygen permeation flux depends on temperature (the purge gas is helium, He flow rate: 60 mL·cm^−2^·min^−1^). (**b**) Effect of CO_2_ concentration on membrane oxygen permeation flux (900 °C, the purge gas is a mixture of helium and CO_2_ and the purge flow rate is 60 mL·cm^−2^·min^−1^).

The oxygen permeation flux was measured using a homemade oxygen permeation test apparatus. As shown in Figure 8a, the oxygen permeation flux of all three membranes increases with the rising operating temperature. Figure 8b illustrates the variation in oxygen permeation flux of the membrane with different CO_2_ concentrations on the purge side (900 °C, CO_2_ concentration: 0–100%).

**Figure 9:**
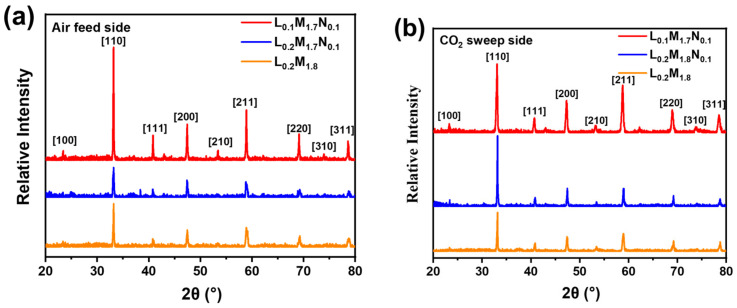
XRD patterns of the high-entropy perovskite disk membrane surfaces after oxygen permeation performance test: (**a**) air feed side; (**b**) CO_2_ sweep side.

Figure 9 shows the XRD diffraction patterns of the disk membranes following the oxygen permeation test under different CO_2_ concentration purge atmospheres.

**Figure 10:**
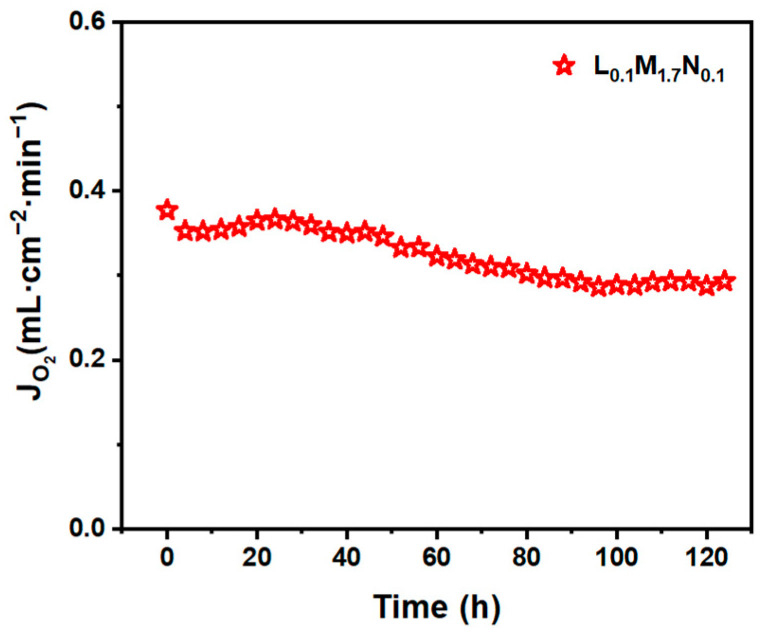
The long-time test of L_0.1_M_1.7_N_0.1_ disk membrane (900 °C purge flow rate: 60 mL·cm^−2^·min^−1^, CO_2_ concentration: 20%).

**Figure 11:**
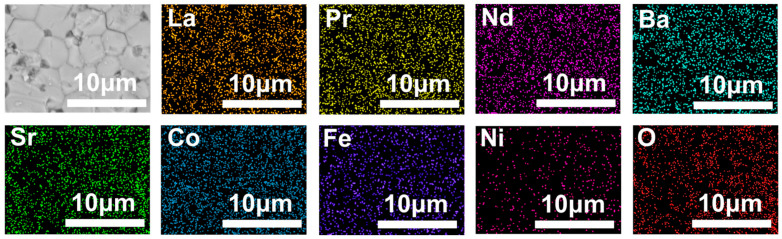
SEM image and corresponding EDS images of CO_2_ purge side after L_0.1_M_1.7_N_0.1_ membrane long-time test.

**Figure 12:**
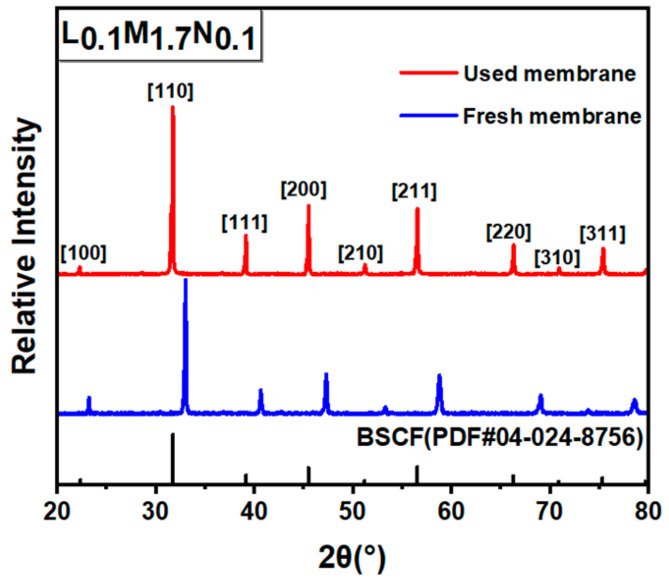
XRD patterns of CO_2_ purge side after L_0.1_M_1.7_N_0.1_ membrane long-time test.

The post-characterization of the L_0.1_M_1.7_N_0.1_ membrane after long-time testing in a 20% CO_2_ purge gas atmosphere is shown in Figure 10, Figure 11 and Figure 12. The result of the long-time test indicated that the L_0.1_M_1.7_N_0.1_ membrane can remain stable in a 20% CO_2_ purge gas atmosphere without a sharp decrease in oxygen permeation flux. The XRD patterns, SEM image, and corresponding EDS images show that the L_0.1_M_1.7_N_0.1_ membrane can maintain an integrated perovskite structure after the long-time test.

## 4. Discussion

The test results indicate that all three high-entropy perovskite oxide membranes exhibit excellent oxygen permeability in a CO_2_-rich atmosphere (the schematic of the oxygen permeation test has been shown in Figure 1). Notably, the L_0.1_M_1.7_N_0.1_ membrane, which incorporates Ni substitution at the B-site and defect design at the A-site, demonstrates the highest oxygen permeability, achieving an oxygen permeation flux of 0.29 mL·min^−1^·cm^−2^ under a pure CO_2_ atmosphere purge. These findings are consistent with various characterization results.

From Figure 2, it is evident that the primary peak position of the BSCF parent material is situated at 31.92°. In contrast, the main peak positions of the high-entropy perovskite materials (HEPMs) are shifted to around 33.11°. This shift is attributed to the unit cell shrinkage resulting from the substitution of larger Ba^2+^ (1.61 Å) and Sr^2+^ (1.44 Å) ions with smaller La^3+^ (1.36 Å), Pr^3+^ (1.32 Å), and Nd^3+^ (1.27 Å) ions at the A-site. Among the HEPMs, the main phase peak of L_0.1_M_1.7_N_0.1_ shifts to a lower angle compared to L_0.2_M_1.8_ and L_0.2_M_1.7_N_0.1_, indicating an expansion of the perovskite lattice. This lattice expansion is due to A-site defects, which cause a loosening of the ABO_3_ framework and benefit oxygen permeation, consistent with reports in the literature [37].

There are apparent differences between the O_2_-TPD and CO_2_-TPD curves of the three HEPMs (the schematic of the O_2_ desorption test system has been shown in Figure 3). This is due to the weak surface oxygen adsorption capacity of the HEPMs, meaning that the surface-adsorbed oxygen (α-O_2_) in the low-temperature region is not apparent [38]. The oxygen desorption behavior observed in the high-temperature region is mainly attributed to the desorption of lattice oxygen and oxygen vacancies (β-O_2_). Compared to L_0.2_M_1.8_, the B-site Ni-substituted materials L_0.2_M_1.7_N_0.1_ and L_0.1_M_1.7_N_0.1_ exhibit higher β-O_2_ desorption peaks at high temperatures. This may be due to the increased oxygen storage capacity in the bulk lattice facilitated by Ni substituting [39]. When Ni²⁺ with a smaller ionic radius substitutes Fe^3+^ or Co^2+^ at the B-site, it converts Fe^3+^ and Co^2+^ into Fe^4+^ and Co^3+^. This process promotes the valence change of metal ions at the B-site and the formation of oxygen vacancies, thereby improving the material’s oxygen storage capacity. When the A-site defect appeared on L_0.1_M_1.7_N_0.1_ material, due to the electrical neutrality the valence state of B-site metal ions increased and created additional oxygen vacancy in the lattice structure. Therefore, the intensity of the β-O_2_ desorption peaks is much higher than that of L_0.2_M_1.8_ and L_0.2_M_1.7_N_0.1_ materials [40]. The peak area ratios are L_0.2_M_1.8_:L_0.2_M_1.7_N_0.1_:L_0.1_M_1.7_N_0.1_ = 1:1.11:4.59. Figure 5b illustrates that the CO_2_ desorption peaks of L_0.2_M_1.7_N_0.1_ and L_0.1_M_1.7_N_0.1_ are markedly smaller than L_0.2_M_1.8_. This indicated that Ni doping could effectively reduce the CO_2_ adsorption capacity of the HEPMs and enhance the operational stability in the CO_2_ atmosphere. The peak area ratios are L_0.2_M_1.8_:L_0.2_M_1.7_N_0.1_:L_0.1_M_1.7_N_0.1_ = 1:0.12:0.46. Therefore, L_0.2_M_1.7_N_0.1_ and L_0.1_M_1.7_N_0.1_ membranes theoretically exhibit higher oxygen permeation flux than L_0.2_M_1.8_ membranes in both normal and CO_2_-rich atmospheres.

The ratios of the O_lat_ and O_ads_ peak areas are proportional to the concentration of oxygen vacancies. The O_lat_/O_ads_ ratios of L_0.2_M_1.8_, L_0.2_M_1.7_N_0.1,_ and L_0.1_M_1.7_N_0.1_ are 0.63:1, 0.64:1, and 0.71:1, respectively. The O_lat_/O_ads_ ratio of the L_0.1_M_1.7_N_0.1_ material is the highest, indicating a higher oxygen vacancy concentration [31,41]. This is consistent with the TPD analysis.

The conduction of three HEPM membranes shows metal-like conductive behavior in the air atmosphere. This phenomenon is attributed to the reduction of B-site cations as temperature rises, leading to a decreased concentration of electric charge carriers. To maintain electrical neutrality, numerous oxygen vacancies are generated in the bulk phase of the material. This phenomenon is observable as a reduction in electric conductivity at the macro scale. The presence of A-site defects in the L_0.1_M_1.7_N_0.1_ material is theoretically associated with increased production of oxygen vacancies [42,43]. According to the charge compensation mechanism, the high-valent cations at the B-site are reduced, decreasing the concentration of high-valent carriers, leading to the lowest electric conductivity of L_0.1_M_1.7_N_0.1_ among the HERMs [43]. Figure 7b shows that the conductivity of three kinds of strip membranes in a CO_2_ atmosphere is lower than in an air atmosphere. This reduction in conductivity may be due to the reaction of B-site ions on the membrane surface with CO_2_, forming carbonates that adhere to the surface and impede electron conduction both on the surface and in the bulk phase. During the test, CO_2_ easily reacted with oxygen vacancies on the surface or inside the perovskites to form carbonates or change the chemical potential of oxygen, affecting the concentration of electrons or holes. When the temperature rose, the mobility of oxygen ions in the perovskite materials increased, and the defect concentration was adjusted, and then the electric conductivity increased. On the other hand, the appearance of carbonates on the surface of the membrane impeded electron conduction. However, when the temperature increased the carbonate decomposed and desorbed, alleviating the effect on conductivity, which manifested as an increase in electric conductivity. So, the conduction of the three HEPM membranes shows semiconductor-like behavior in the CO_2_ atmosphere.

Across the temperature range tested, the L_0.1_M_1.7_N_0.1_ membrane exhibits the highest oxygen permeation performance. This observation is consistent with the O_2_-TPD and XPS characterization results, suggesting that introducing A-site oxygen vacancies in high-entropy perovskite oxides enhances oxygen permeation performance. In actual industrial applications, such as oxygen-enriched combustion, CO_2_-rich gas is often recycled as a purge gas and heat source [44,45]. Therefore, the oxygen permeability and stability of the material in a CO_2_-rich atmosphere are of significant importance. Figure 8b demonstrates that the oxygen permeation flux of all three materials decreases with increasing CO_2_ concentration. This decrease may be attributed to the formation of a continuous carbonate layer by A-site alkaline earth metal ions on the membrane surface in a CO_2_ atmosphere, which impedes the transmission of oxygen ions. Furthermore, CO_2_ adsorption on the membrane surface reduces the number of active sites for oxygen permeation, leading to diminished performance. After the pure CO_2_ atmosphere test, the membranes were tested in a He atmosphere, resulting in varying degrees of improvement in oxygen permeation flux, surpassing the flux observed in the pure He purge test (CO_2_ concentration = 0). This behavior aligns with the reported performance of BSCF materials after CO_2_ purging [46]. It indicates that the reduction in oxygen flux with increasing CO_2_ purge gas concentration may primarily be due to the adsorption of CO_2_ on the membrane surface. Compared to materials L_0.2_M_1.8_ and L_0.2_M_1.7_N_0.1_, the L_0.1_M_1.7_N_0.1_ membrane exhibits higher oxygen permeation flux under all CO_2_ atmospheres. This may be because the L_0.1_M_1.7_N_0.1_ material has the highest oxygen vacancy concentration and a lower carbon dioxide adsorption capacity on the membrane surface. Under pure CO_2_ purge conditions, the oxygen permeation flux of the L_0.1_M_1.7_N_0.1_ membrane reaches 0.29 mL·min^−1^·cm^−2^. In contrast, the oxygen flux of the L_0.2_M_1.8_ and L_0.2_M_1.7_N_0.1_ membranes are 0.23 mL·min^−1^·cm^−2^ and 0.20 mL·min^−1^·cm^−2^, respectively. The oxygen permeation flux of the BSCF membrane reported in the literature is nearly 0 under the 20% CO_2_ atmosphere [46], indicating that the high-entropy design of the BSCF material significantly enhances its ability to resist CO_2_. Moreover, the Ni substitution at the B-site and the introduction of A-site defects further optimize the oxygen permeability of the HEPM membranes. As shown in Figure 9a,b, all three membranes maintain a good perovskite phase structure after the test, with no evidence of carbonate formation. This indicates that the L_0.2_M_1.8_, L_0.2_M_1.7_N_0.1,_ and L_0.1_M_1.7_N_0.1_ membranes exhibit excellent structural stability and CO_2_ corrosion resistance after high-entropy design.

In practical applications, the operational stability of the membrane under specific conditions is an important performance parameter. This work selected the L_0.1_M_1.7_N_0.1_ membrane with a high oxygen permeation flux for stability testing. As shown in Figure 10, during a 120 h stability test, the oxygen permeability remained stable at approximately 0.3 mL·min^−1^·cm^−2^, indicating that the L_0.1_M_1.7_N_0.1_ membrane exhibits substantial operational stability under CO_2_-rich conditions. The membrane after the long-time test was characterized by SEM and corresponding EDS. As shown in Figure 11, the CO_2_ purge side of the membrane can still maintain a good grain boundary structure, and the corrosion phenomenon is not obvious. The EDS images exhibited that there was no carbonate appearance on the membrane surface. Furthermore, the XRD patterns of the membrane after the long-time test, as shown in Figure 12, confirmed that the membrane still maintained a good perovskite phase structure, without carbonate peak appearance. The peaks shifted to low degrees due to the lattice expansion caused by the thermal reduction and spin state transition of the B-site ions during the long-time test. These results demonstrate that the L_0.1_M_1.7_N_0.1_ high-entropy perovskite material can be operated stably for a long time under a 20% CO_2_ purge atmosphere.

## 5. Conclusions

In this study, we synthesized La_0.2_Pr_0.2_Nd_0.2_Ba_0.2_Sr_0.2_Co_0.7_Fe_0.2_Ni_0.1_O_3-δ_ (L_0.2_M_1.7_N_0.1_) materials by introducing a Ni substitution strategy at the B-site, building upon the La_0.2_Pr_0.2_Nd_0.2_Ba_0.2_Sr_0.2_Co_0.8_Fe_0.2_O_3-δ_ high-entropy perovskite framework with good CO_2_ stability. Subsequently, we developed La_0.1_Pr_0.2_Nd_0.2_Ba_0.2_Sr_0.2_Co_0.7_Fe_0.2_Ni_0.1_O_3-δ_ (L_0.1_M_1.7_N_0.1_) materials by defect design at the A position of L_0.2_M_1.7_N_0.1_ materials to enhance oxygen permeation flux. The results indicate that all three materials exhibit good oxygen permeation flux in a CO_2_-rich atmosphere, among which the oxygen permeation flux of the L_0.1_M_1.7_N_0.1_ membrane in a pure CO_2_ atmosphere can reach 0.29 mL·min^−1^·cm^−2^. To explore the practical prospects, we conducted a 120 h stability test on the L_0.1_M_1.7_N_0.1_ membrane in a 20% CO_2_ atmosphere on the purge side. The test results demonstrate that the oxygen permeation flux remained stable at approximately 0.3 mL·min^−1^·cm^−2^, and the SEM images of the membrane post-test showed clear grain boundaries with no additional phases generated in the XRD diffraction pattern. The findings indicate that A-site-defective HEPMs demonstrate significant potential for CO_2_ capture, storage, and utilization applications.

## Figures and Tables

**Table 1:** Goldschmidt tolerance factor (t), size difference of cations (δ), and mixed entropy (ΔSmix) of the materials.

Materials	t	δ(a)	δ(b)	ΔS_mix_
L_0.2_M_1.8_	0.982	8.48%	6.72%	1.98R
L_0.2_M_1.7_N_0.1_	0.978	8.48%	4.11%	2.04R
L_0.1_M_1.7_N_0.1_	0.930	8.89%	4.11%	1.99R

## Data Availability

The data presented in this study are available upon reasonable request from the corresponding author due to privacy.
